# Comparison of SPECT/CT and MRI in Diagnosing Symptomatic Lesions in Ankle and Foot Pain Patients: Diagnostic Performance and Relation to Lesion Type

**DOI:** 10.1371/journal.pone.0117583

**Published:** 2015-02-10

**Authors:** Seunggyun Ha, Sung Hwan Hong, Jin Chul Paeng, Dong Yeon Lee, Gi Jeong Cheon, Amitabh Arya, June-Key Chung, Dong Soo Lee, Keon Wook Kang

**Affiliations:** 1 Department of Nuclear Medicine, Seoul National University Hospital, Seoul, Korea; 2 Department of Radiology, Seoul National University Hospital, Seoul, Korea; 3 Department of Orthopedic Surgery, Seoul National University Hospital, Seoul, Korea; 4 Department of Nuclear Medicine, Sanjay Gandhi Postgraduate Institute of Medical Sciences, Lucknow, India; Institute of Automation, Chinese Academy of Sciences, CHINA

## Abstract

**Purpose:**

The purpose of this study was to compare the diagnostic performance of SPECT/CT and MRI in patients with ankle and foot pain, with regard to the lesion types.

**Materials and Methods:**

Fifty consecutive patients with ankle and foot pain, who underwent ^99m^Tc-MDP SPECT/CT and MRI, were retrospectively enrolled in this study. Symptomatic lesions were determined based on clinical examination and response to treatment. On MRI and SPECT/CT, detected lesions were classified as bone, ligament/tendon, and joint lesions. Uptake on SPECT/CT was assessed using a 4-grade system. Sensitivity, specificity, positive predictive value (PPV) and negative predictive value (NPV) of SPECT/CT and MRI were evaluated in all detected lesions and each lesion type. Diagnostic value of uptake grade was analyzed using receiver-operating characteristics (ROC) curve analysis, and diagnostic performance was compared using Chi-square or McNemar tests.

**Results:**

In overall lesions, the sensitivity, PPV and NPV of SPECT/CT for symptomatic lesions were 93%, 56%, 91%, and they were 98%, 48%, 95% for MRI. There was no significant difference between SPECT/CT and MRI. However, the specificity of SPECT/CT was significantly higher than that of MRI (48% versus 24%, *P* = 0.016). Uptake grade on SPECT/CT was significantly higher in symptomatic lesions (*P* < 0.001), and its area under curve on ROC analysis was 0.787. In the analysis of each lesion type, the specificity of SPECT/CT was poor in joint lesions compared with other lesion types and MRI (*P* < 0.001, respectively). MRI exhibited lower specificity than SPECT/CT in bone lesions (*P* = 0.004) and ligament/tendon lesions (*P* < 0.001).

**Conclusions:**

SPECT/CT has MRI-comparable diagnostic performance for symptomatic lesions in ankle and foot pain patients. SPECT/CT and MRI exhibit different diagnostic specificity in different lesion types. SPECT/CT may be used as a complementary imaging method to MRI for enhancing diagnostic specificity.

## Introduction

In diagnosing ankle and foot pain, clinical examination is a basic process and may provide accurate diagnosis in many cases. However, clinical examination is often limited, particularly in patients with vague and chronic pain. Imaging studies are effective for diagnosis and planning of surgical treatment. Currently, magnetic resonance imaging (MRI) is the most effective diagnostic imaging method in ankle and foot pain, with strengths such as high image resolution and excellent soft tissue contrast. In an expert consensus report, MRI was recommended as the most appropriate imaging method for ankle and foot pain when simple X-ray images are negative [1]. However, clinical MRI is based on structural changes, and may have limitations when there are only subtle structural changes or when there are several coexistent lesions [2,3].

Bone scan and single photon emission computed tomography (SPECT) using ^99m^Tc-labeled phosphonate agents can show increased bone metabolism or bone turnover in injured bones, which is also related to the joint inflammation and ligament/tendon injury as a secondary change [4]. For detection of pathogenic lesions, changes in bone turnover are often complementary to structural changes. Thus, bone scan and SPECT have been demonstrated to be effective in diverse musculoskeletal disorders. Furthermore, SPECT/CT, a hybrid imaging method incorporating SPECT and computed tomography (CT), has the advantage that high-resolution CT is utilized for lesion localization [5]. Bone SPECT/CT has been reported to have a higher diagnostic value than conventional planar bone scan or SPECT alone [6–9]. Although there have been only few studies on direct comparison of SPECT/CT and MRI in musculoskeletal disorders, a recent study reported that SPECT/CT has a higher specificity with a relatively lower sensitivity than MRI in cases with nonspecific hand and wrist pain [2]. In cases with ankle and foot pain, bone SPECT/CT is also expected to be effective and of an additive diagnostic value to MRI, because of their different imaging mechanism [10]. For this purpose, further understanding is required in terms of various diagnostic characteristics of each imaging method.

In this study, we investigated the diagnostic performance of SPECT/CT and MRI in cases with ankle and foot pain. We also analyzed the influence of lesion type on the diagnostic performance of SPECT/CT and MRI.

## Materials and Methods

### Patients

The study design was approved by the Institutional Review Board (IRB) of Seoul National University Hospital, and informed consent from each patient was waived by the approving IRB.

From June 2011 to January 2013, consecutive patients who underwent SPECT/CT for ankle and foot pain were retrospectively included in this study. Among these patients, those who underwent both SPECT/CT and MRI within 3 months from each other were selected. Patients who received any surgery in the ankle and foot within 3 months before either SPECT/CT or MRI were excluded due to possible postoperative reactive changes. Finally, 50 patients were included in the analysis, and the patient records and information were anonymized and de-identified prior to analysis.

### Image Acquisition

Patients were injected with 740 MBq ^99m^Tc-methylene diphosphonate (MDP) and images were acquired 4 hours later. SPECT/CT was performed using a hybrid SPECT/CT scanner combining a dual-head gamma camera and a 16-slice CT scanner (Discovery NM/CT 670, GE Healthcare, Waukesha, WI, US). The gamma camera was equipped with low-energy high-resolution collimators, and 60 step-and-shoot images (3° intervals) were acquired by each detector for 23 seconds per step. Energy window was open by ± 10% with centered at 141 keV. SPECT images were reconstructed on 128×128 matrices using an iterative algorithm of ordered-subset expectation maximization (iteration 8, subsets 8). After SPECT acquisition, a helical CT scan for attenuation correction and anatomical localization was performed without using contrast agent (100 kVp, 100 mAs, and pitch 0.938) and images were reconstructed into 1.25-mm slices.

In 50 patients, 24 MRI scans were performed using 3.0-T scanners (Magnetom Verio/Trio, Siemens Medical Solution, Erlangen, Germany) and 26 scans were performed using various outside MRI units. Outside MRI scans were performed using a 3.0-T scanner in one patient, and using 1.5-T scanners in 24 patients. In one patient, MRI scan was performed using a 1.0-T scanner and included in the analysis after approval of the acceptable image quality. In all patients, spin echo T1- and T2-weighted images and fat-suppressed fluid-sensitive images were obtained. The imaging planes included at least two transverse, one sagittal and one coronal planes. Gadolinium-enhanced images were performed in two patients.

### Image Interpretation and Analysis of Diagnostic Performance

All original image files of MRI were transferred to our picture archiving and communication system (Maroview, Seoul, Korea) and reviewed by an experienced radiologist specialized in musculoskeletal imaging (S.H.H.), who was blinded to SPECT/CT findings. All abnormal findings were analyzed in terms of fracture, osteochondral lesion, accessory bone, bone marrow edema, cartilage injury, ligament or tendon tear, and arthritis. SPECT/CT images were displayed in axial, coronal, and sagittal planes on an image-analysis workstation (Xeleris 3, GE Healthcare, Waukesha, WI, US). Images were interpreted and graded based on the consensus of three experienced nuclear medicine physicians (S.H., J.C.P., and G.J.C.) blinded to MRI findings. An abnormally increased uptake compared with background was regarded as a lesion, and the location of a lesion was determined based on co-registered CT images. For semi-quantitative analysis, uptake was assessed using a 4-grade system (0, none; 1, mild; 2, moderate; 3, severe).

All lesions detected on SPECT/CT and/or MRI were included in the analysis. An experienced orthopedic surgeon (D.Y.L.) who specialized in foot and ankle disorders reviewed all lesions, after treatment based on clinical and image findings and appropriate follow-up (median 3.2 months, range 0.3–14.3 months). Final clinical diagnosis was determined by complete clinical examination, thorough assessment of discomfort and response to treatment after follow-up. In 24 patients, diagnosis was confirmed by direct evaluation during arthroscopic surgery such as chondroplasty, ligament repair, and bony fragment removal. In clinical diagnosis, pain sites were classified into ankle, forefoot, midfoot, and hindfoot. Specific locations of pain and tenderness were also marked. When a site of pain or tenderness was matched with specific abnormality on images, it was determined as a correct pathologic site. Final diagnosis was made based on these image findings, history of trauma, and related systemic symptoms.

**Fig 1:**
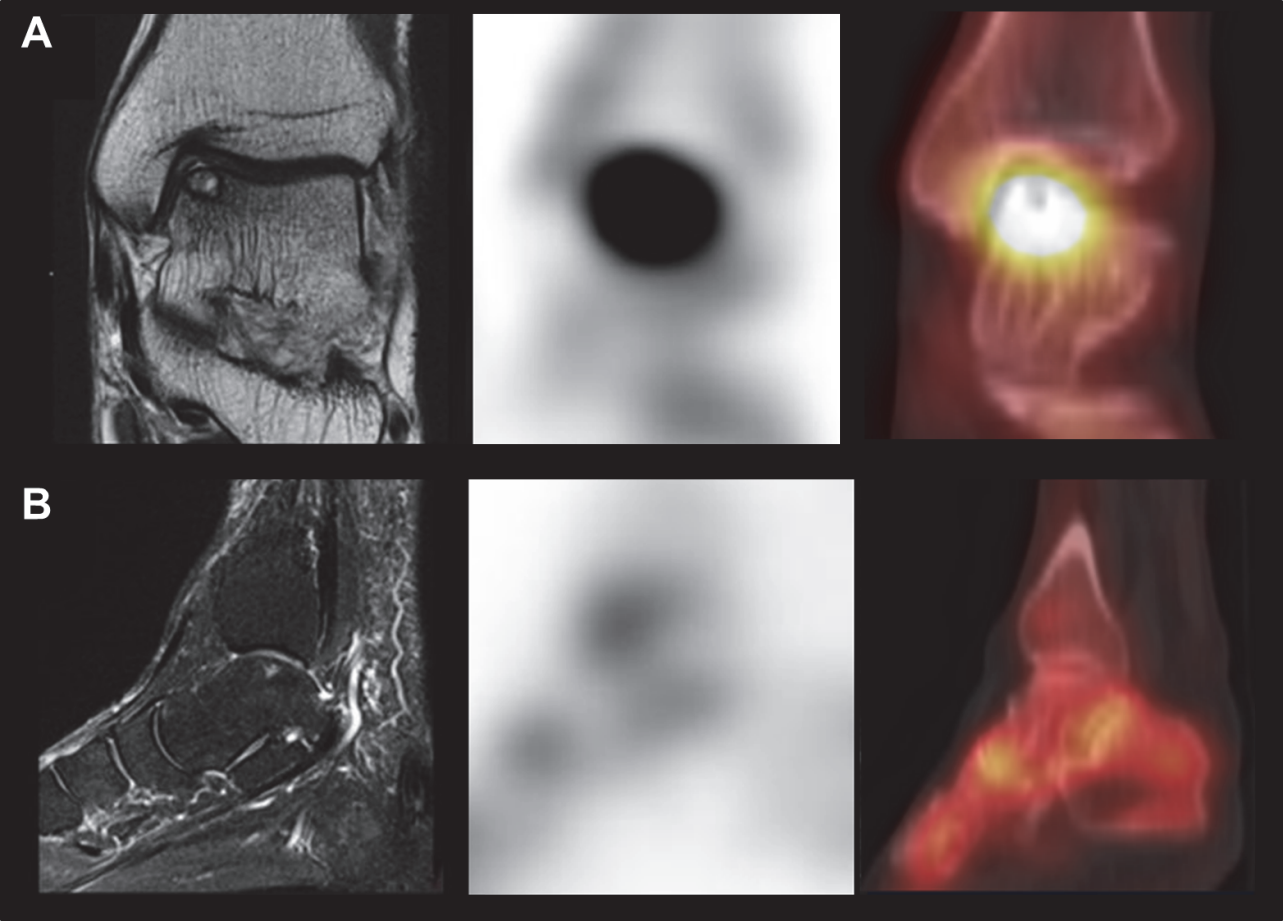
Images of representative cases. (A) Axial MRI (left) of a19-year-old woman revealed an osteochondral lesion in the right medial talar dome area. SPECT/CT (middle and right) also exhibited focal intense uptake in the same area, concordant with MRI. The lesion was diagnosed with symptomatic osteochondral lesion of talus, and her ankle pain improved after arthroscopic multiple drilling of the talus. (B) Sagittal fat-saturated MRI (left) of a 65-year-old woman with posterior heel pain revealed an osteochondral lesion in the right medial talar dome area, whereas SPECT/CT showed no abnormal uptake in the same area. The pain improved after surgical removal of a right calcaneal bony fragment, and the talar dome lesion was determined to be asymptomatic.

Based on the final diagnosis, lesions on the images were classified as symptomatic (related to the final diagnosis and presumably causing symptoms) or asymptomatic (Fig. 1). Image findings for symptomatic lesions were regarded as true-positive, and the diagnostic performance of SPECT/CT and MRI was evaluated in terms of sensitivity, specificity, positive predictive value (PPV) and negative predictive value (NPV).

### Diagnostic Performance According to Lesion Type

**Fig 2:**
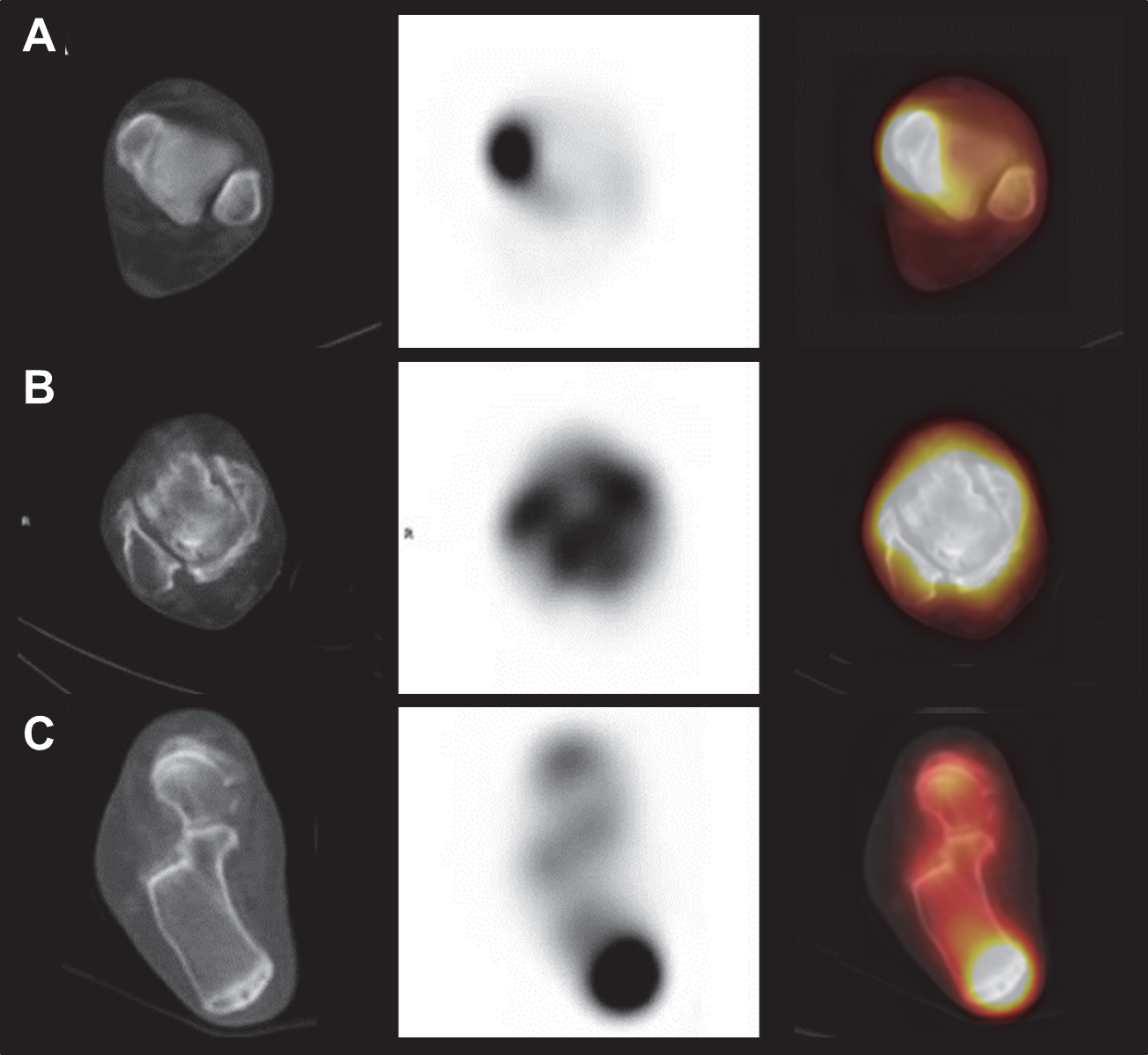
Representative images of lesion types. CT-axial (left), SPECT-axial (middle), and fusion-axial (right) images of SPECT/CT are shown; a bone lesion (contusion) involving the medial malleolus (A), a joint lesion (arthritis) involving the left talocrural joint (B), and a ligament/tendon lesion (tendinitis) involving the Achilles tendon insertion site of calcaneus (C).

For comparing the diagnostic characteristics of SPECT/CT and MRI in terms of lesion type, lesions were classified into three groups; (1) bone lesions such as osteochondral lesion of talus (OLT), bony fragment or accessory bone, bone contusion, and fracture; (2) ligament/tendon lesions such as tear or inflammation in medial ligament complex (including posterior tibialis tendon), lateral ligament complex (including anterior tibiofibular ligament and calcaneofibular ligament), Achilles tendon, and plantar fascia; and (3) joint lesions, such as osteoarthritis, rheumatoid arthritis, and non-specified arthritis which was detected on SPECT/CT without discernible structural change. On SPECT/CT, uptake in a single bone was classified as a bone lesion and uptake in an attachment site of a specific ligament or tendon was classified as a ligament/tendon lesion. Uptake along a joint surface was classified as a joint lesion. Representative SPECT/CT images of each group are shown in Fig. 2. Diagnostic performance of SPECT/CT and MRI was assessed in each lesion type.

### Statistical Analysis

All data were expressed as mean ± standard deviation. Chi-square test and McNemar test were used for comparing diagnostic performance. Semi-quantitative grades were compared between groups using Mann-Whitney U-test or Kruskal-Wallis test. Receiver-operating characteristics (ROC) curve analysis was performed for diagnostic power of semi-quantitative grade on SPECT. *P*-values less than 0.05 were regarded significant. All statistical analyses were performed using commercial software packages of SPSS (Version 18.0, IBM Software, Chicago, IL, US) and MedCalc (Version 12.2.1.0, MedCalc Software, Ostend, Belgium).

## Results

### Patient Characteristics

Among 50 patients, 28 were male and 22 were female. Median age was 46 years (range 16–75 years). The median time interval between SPECT/CT and MRI was 30.5 days (range 0–87 days). Forty patients underwent SPECT/CT after MRI, and 10 patients underwent SPECT/CT before MRI. As two patients had bilateral ankle and foot pain, a total of 52 cases were included in the analysis. A total of 147 lesions were detected on either SPECT/CT or MRI, and 61 lesions were determined to be symptomatic in the final diagnosis; 37 were bone lesions, 14 were ligament/tendon lesions, and 10 were joint lesions, in terms of lesion types.

In 24 patients, 27 symptomatic lesions were diagnosed by direct evaluation during surgery and postsurgical follow-up; clinical diagnosis was made for 34 lesions. For ankle pain, 11 were diagnosed with OLT based on pain and image findings. Eight lesions were diagnosed with ligament sprains involving medial or lateral ligament complexes, based on chronic medial or lateral ankle pain, tenderness, and joint instability. Additionally, there was no abnormality on images except sprain-related findings. Three lesions were diagnosed with systemic inflammatory arthritis including ankylosing spondylitis based on multiple joint involvements, synovitis finding on MRI, and high ESR levels. Other ankle pain lesions were diagnosed with osteoarthritis and traumatic arthritis based on image findings and patient history. Forefoot and midfoot pain lesions were diagnosed with stress fracture of metatarsal bone, traumatic arthritis in metatarsophalangeal joint, bony contusions in distal tibia and navicular bone, tarsal coalition, and intertarsal joint synovitis, which were based on pain-matched image findings. Hindfoot pain lesions were diagnosed with os trigonum syndrome and Achilles tendinitis, based on posterior heel pain and related image findings. The final clinical diagnoses are summarized in Table 1.

**Table 1:** Final diagnosis for symptomatic lesions.

Lesion type	Final diagnosis	Surgically diagnosed	Clinically diagnosed: pain site				Sum
			Ankle	Forefoot	Midfoot	Hindfoot	
Bone	Osteochondral lesion of talus	19	11				30
	Bony fragment or accessory bone	3				1	4
	Bone contusion and secondary change				2		2
	Fracture			1			1
Ligament/tendon	Sprain	1	8	1			10
	Achilles tendinitis	3				1	4
Joint	Osteoarthritis	1	2				3
	Systemic inflammatory arthritis		3				3
	Arthritis, others		1	1	2		4
	Total	27	25	3	4	2	61

### Diagnostic Performance for Symptomatic Lesions

**Fig 3:**
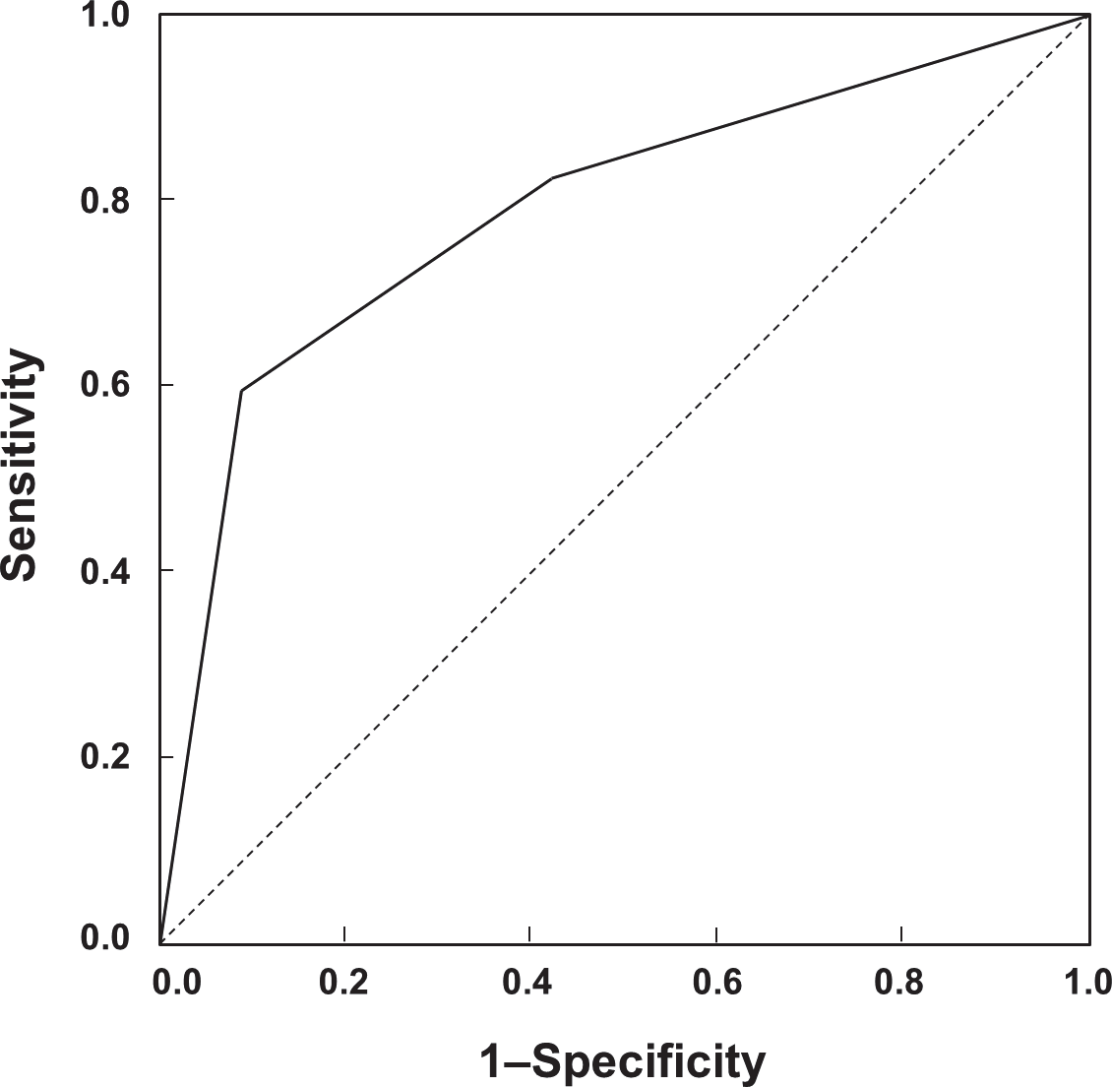
ROC curve analysis of uptake grade for diagnosing symptomatic lesions. The area under curve was 0.787, and the optimal cutoff was grade 3 with 60% sensitivity and 91% specificity.

On SPECT/CT, 102 lesions with increased uptake were detected, 57 of which were symptomatic in the final diagnosis. The sensitivity, specificity, PPV and NPV of SPECT/CT for symptomatic lesions were 93%, 48%, 56% and 91%, respectively. In the semi-quantitative analysis, uptake of grades 1, 2, and 3 was observed in 38, 26, and 38 lesions respectively. The mean uptake grade was significantly higher for symptomatic lesions than asymptomatic lesions (2.4 ± 0.8 *versus* 1.5 ± 0.7, *P* < 0.001; Table 2). The PPV for symptomatic lesions was 29% (11/38) for grade 1, 46% (12/26) for grade 2, and 89% (34/38) for grade 3 uptake. The PPV of grade 3 uptake was significantly higher than that of grade 1 and grade 2 uptake (*P* < 0.001 and *P* = 0.005, respectively). The ROC curve analysis revealed a high area under curve (0.787), and the optimal sensitivity and specificity were 60% and 91%, respectively, at the cutoff of grade 3 (Fig. 3). Although 11 bone lesions without increased uptake were additionally detected on co-registered CT, all were asymptomatic and not included in the analysis.

**Table 2:** Uptake grade on SPECT/CT.

	Uptake grade	*P*
Correlation with symptoms		
*Symptomatic*	2.4 ± 0.8	< 0.001
*Asymptomatic*	1.5 ± 0.7	
Lesion type		
*Bone*	2.3 ± 0.8	0.002
*Ligament/tendon*	1.6 ± 0.9	
*Joint*	1.8 ± 0.8	

On MRI, 125 lesions were detected, and 60 of them were symptomatic. The sensitivity, PPV and NPV of MRI for symptomatic lesions were 98%, 48%, and 95%, respectively, and these values did not differ significantly from those of SPECT/CT (*P* = 0.371, 0.858 and 0.681, respectively). However, the specificity of MRI was 24% and it was significantly lower than that of SPECT/CT (*P* = 0.016).

Out of the 147 lesions detected by either MRI or SPECT/CT, 46 were detected by MRI only, 21 by SPECT/CT only, and 80 by both imaging methods. The concordant detection rate was 54% (80/147). However, for symptomatic lesions, one was detected by SPECT/CT only, 4 by MRI only, and 56 by both imaging methods. The concordant detection rate of symptomatic lesions was 92% (56/61); significantly higher than that of overall lesions (*P* < 0.001).

### Diagnostic Performance According to Lesion Type

The numbers of lesions in each lesion type are shown in Table 3. Uptake grade was significantly higher in bone lesions than in other lesions (*P* = 0.002, Table 2). Asymptomatic lesions detected by SPECT/CT were mostly joint lesions, whereas those detected by MRI were mostly ligament/tendon lesions.

**Table 3:** Number of lesions detected on each imaging method according to lesion type.

	Bone	Ligament/tendon	Joint	Total
SPECT/CT				
*Symptomatic*	37	10	10	57
*Asymptomatic*	9^†^	11	25	45
MRI				
*Symptomatic*	37	14	9	60
*Asymptomatic*	19	37	9	65

^†^11 lesions were additionally detected by combined CT only, with grade 0 uptake

The diagnostic performance for symptomatic lesions in each lesion type is shown in Table 4. SPECT/CT exhibited a relatively poor sensitivity for ligament/tendon lesions than other lesion types (71% *versus* 100%). SPECT/CT exhibited a poor specificity for joint lesions (0%), which was significantly lower than that of bone lesions and ligament/tendon lesions (*P* < 0.001, respectively), and also lower than that of MRI (*P* < 0.001). MRI exhibited high sensitivity in all lesion types; 100% in bone lesions and ligament/tendon lesions, and 90% in joint lesions. However, MRI exhibited lower specificity than SPECT/CT in bone lesions (*P* = 0.004) and ligament/tendon lesions (*P* < 0.001).

**Table 4:** Diagnostic performance of SPECT/CT and MRI for symptomatic lesions in each lesion type.

	Bone (N = 58)	Ligament/tendon (N = 54)	Joint (N = 35)	Total
SPECT/CT				
Sensitivity	100%	71%	100%	93%
	(37/37)	(10/14)	(10/10)	(57/61)
Specificity	57%	73%	0%	48%
	(12/21)	(29/40)	(0/25)	(41/86)
PPV	80%	48%	29%	56%
	(37/46)	(10/21)	(10/35)	(57/102)
NPV	100%	88%	N/A	91%
	(12/12)	(29/33)		(41/45)
MRI				
Sensitivity	100%	100%	90%	98%
	(37/37)	(14/14)	(9/10)	(60/61)
Specificity	10%	8%	64%	24%
	(2/21)	(3/40)	(16/25)	(21/86)
PPV	66%	27%	50%	48%
	(37/56)	(14/51)	(9/18)	(60/125)
NPV	100%	100%	94%	95%
	(2/2)	(3/3)	(16/17)	(21/22)

*PPV* positive predictive value, *NPV* negative predictive value, *N/A* not applicable

## Discussion

In this study, we compared diagnostic performance of SPECT/CT and MRI in ankle and foot pain patients. The sensitivity, PPV, and NPV of SPECT/CT for symptomatic lesions were comparable to those of MRI. However, SPECT/CT exhibited a higher specificity than MRI, in bone and ligament/tendon lesions.

In patients with ankle and foot pain, it is crucial to detect the lesion responsible for the pain, for determining adequate treatment. MRI, in combination with other radiologic images, is currently regarded as the most appropriate diagnostic imaging for benign bone and soft tissue lesions [1]. However, because usual MRI is based on structural changes, it has a limitation with regard to specifically determining the pathogenic lesions responsible for the pain. Structural abnormality is often observed as sequelae of old trauma as well as anatomical variants, irrespective of pain [11]. Besides, pathology with clinical significance is not always visualized as structural change. These hurdles bring about high level of difficulty for interpretation of musculoskeletal MRI scan and may lead to high inter-observer variation [12]. Also in this study, MRI detected many asymptomatic lesions, which impaired the diagnostic specificity of MRI. Thus, bone SPECT/CT that facilitate visualization of bone metabolism may be used as a complementary imaging method [13–15], because increased uptake on bone SPECT/CT is closely correlated with pathologic bone turnover [16].

On bone scans using ^99m^Tc-phosphonate agents such as MDP, dicarboxypropane diphosphonate, and hydroxymethylene diphosphonate, the uptake reflects osteoblastic activity or bone turnover that may be increased in response to inflammation of the bone. Additionally, uptake is also increased in cases of arthritis and injury of the ligament and tendon, as a secondary change [17–19]. The usefulness of bone scan in evaluating severity of arthritis and diagnosing early arthritis has been repeatedly reported in clinical field [20,21]. Additionally, a previous study has demonstrated that symptomatic tendon tear presented higher uptake on bone scan than asymptomatic tendon tear [22]. Thus, bone scan may enhance the sensitivity for lesions without structural change and the specificity for lesions with asymptomatic incidental structural change. Pathologic uptake on SPECT is also well correlated with symptoms and can affect decisions with regard to the treatment plan [3,23–27].

However, bone scan has a low spatial resolution for lesion localization in complex anatomical structures such as the ankle and foot. A hybrid imaging of SPECT/CT can provide high-resolution CT images for lesion localization, and enhanced diagnostic accuracy [9,28,29]. In small lesions of the hand and wrist, SPECT/CT demonstrated a higher specificity than MRI for causative pathology [2]. SPECT/CT was also reportedly useful for localizing active degenerative disease in small joints of ankle [8,30]. Additionally, uptake on bone SPECT/CT was significantly associated with pain or pain relief after local injection of anesthetics [25,27]. In OLT, SPECT/CT results were significantly correlated with the severity of pain [23,31], and ^99m^Tc-MDP SPECT/CT results led to changes in treatment plan in 48–52% of patients, compared with MRI alone [3].

In the present study, we investigated the diagnostic performance of SPECT/CT and MRI in ankle and foot pain patients, and found that the sensitivity, PPV and NPV of SPECT/CT for symptomatic lesions were comparable to those of MRI. Furthermore, SPECT/CT exhibited a significantly higher specificity than MRI. Both SPECT/CT and MRI had high sensitivity for symptomatic lesions, and the concordant detection rate was 92% for symptomatic lesions whereas it was 54% for all detected lesions regardless of symptoms.

Quantification is one of the strengths of nuclear imaging methods including SPECT. In the present study, there was a significant difference between symptomatic and asymptomatic lesions in semi-quantified uptake grades on SPECT/CT. Additionally, ROC curve analysis exhibited a considerable diagnostic power of uptake grade for diagnosing symptomatic lesion. Thus, the severity of uptake may be regarded as a marker for significance of a lesion. Additionally, CT images on SPECT/CT can be used for diagnosis. In the present study, a full diagnostic CT was performed and a total of 11 accessory bones were detected on CT, most of which were also detected by MRI. Although we did not include abnormal CT findings in the analysis for detection of symptomatic lesion, diagnostic performance could be further enhanced by using CT images.

Intriguingly, SPECT/CT and MRI exhibited a tendency in lesion detection with regard to lesion types. SPECT/CT exhibited a poor specificity in joint lesions whereas the specificity of MRI was relatively poor in bone and ligament/tendon lesions. It is a logical result for ligament/tendon lesions because MRI can sensitively detect the lesions by direct visualization of injury, whereas SPECT/CT can detect the lesions only after they induce a secondary stress change at the bony insertion sites. On the other hand, SPECT/CT detected many asymptomatic uptakes in the small joints. Although it has been reported that mild arthritis is sensitively detected on SPECT [8,30], there is no consensus that SPECT detects arthritis more sensitively than MRI. In the present study, there were 16 joint lesions with increased uptake on SPECT/CT. But they were not related to structural change on non-enhanced MRI and most of them were asymptomatic. It may be regarded as subclinical arthritis.

Based on the results of present study, SPECT/CT could be suggested as an effective complementary imaging method for MRI in ankle and foot pain patients. SPECT/CT may be effective for improving diagnostic specificity in combination with MRI. In this study, when a lesion was deemed to be positive in case both SPECT/CT and MRI were positive, the specificity was markedly improved with relatively small loss of sensitivity particularly in bone and ligament/tendon lesions. Thus, SPECT/CT may be helpful in patient selection for surgical treatment, when multiple lesions are detected on MRI. Additionally, SPECT/CT could be effectively used as an alternative imaging method for MRI when MRI is not easily accessible due to high cost or patient condition. Although there is some concern about additional radiation exposure from CT component of SPECT/CT, the radiation dose of CT to the extremity is relatively small because the extremities are not radiation-sensitive organs. Effective dose from unilateral ankle and foot CT has been reported to be 0.07 ± 0.05 mSv at 120 kVp and 143 mA [32]. Thus, overall radiation exposure from ankle and foot SPECT/CT is estimated to be approximately 5.5 mSv, which is acceptable in terms of risk-benefit balance.

This study has several limitations. First, SPECT/CT was not performed for all patients with ankle and foot pain in our institution due to retrospective study design. Thus, the patient population included in our study may have been heterogeneous and biased. Second, we included all lesions detected by either SPECT/CT or MRI without comprehensive evaluation of lesions. This method may have underestimated the specificity of SPECT/CT or MRI. Third, MRI protocol was heterogeneous by including outside MRI. Most of the outside MRI scans were obtained using 1.5-T scanners whereas 3.0-T scanners were used in our institution. However, performance of 1.5-T MRI scanners is usually enough for diagnosing acute bone stress change and stress-induced injuries in the foot [33], and image quality of all MRI was confirmed by experienced radiologists. Finally, the SPECT/CT and MRI findings were not blinded in determining final diagnosis of symptomatic lesions, although the final diagnosis was based on clinical findings and follow-up results. This limitation is a practical inevitability, because final diagnoses for the cause of ankle and foot pain cannot be made based on clinical findings alone, in current clinical practice.

## Conclusions

In ankle and foot pain patients, SPECT/CT has diagnostic performance for symptomatic lesions which is comparable to those of MRI. SPECT/CT and MRI exhibit different diagnostic specificity in different lesion types. Thus, SPECT/CT may be used as a complementary imaging to MRI in ankle and foot pain patients, particularly for enhancing diagnostic specificity.

## Supporting Information

S1 Fig A case of bone lesion: osteochondral lesion of talus. (TIF)

S2 Fig A case of ligament/tendon lesion: anterior talo-fibular ligament injury. (TIF)

S3 Fig A case of joint lesion: intertarsal joint arthritis. (TIF)

## References

[1] DeSmet AA, Dalinka MK, Alazraki N, Berquist TH, Daffner RH, et al. (2000) Chronic ankle pain. American College of Radiology. ACR Appropriateness Criteria. Radiology 215 Suppl: 321–332. 11037444

[2] Huellner MW, Burkert A, Schleich FS, Schurch M, Hug U, et al (2012) SPECT/CT versus MRI in patients with nonspecific pain of the hand and wrist—a pilot study. Eur J Nucl Med Mol Imaging 39: 750–759. 10.1007/s00259-011-2034-3 22237845

[3] Leumann A, Valderrabano V, Plaass C, Rasch H, Studler U, et al (2011) A novel imaging method for osteochondral lesions of the talus: comparison of SPECT-CT with MRI. Am J Sports Med 39: 1095–1101. 10.1177/0363546510392709 21300809

[4] Papathanassiou D, Bruna-Muraille C, Jouannaud C, Gagneux-Lemoussu L, Eschard JP, et al (2009) Single-photon emission computed tomography combined with computed tomography (SPECT/CT) in bone diseases. Joint Bone Spine 76: 474–480. 10.1016/j.jbspin.2009.01.016 19800831

[5] Mariani G, Bruselli L, Kuwert T, Kim EE, Flotats A, et al (2010) A review on the clinical uses of SPECT/CT. Eur J Nucl Med Mol Imaging 37: 1959–1985. 10.1007/s00259-010-1390-8 20182712

[6] Linke R, Kuwert T, Uder M, Forst R, Wuest W (2010) Skeletal SPECT/CT of the peripheral extremities. AJR Am J Roentgenol 194: W329–335. 10.2214/AJR.09.3288 20308478

[7] Zhang Y, Shi H, Gu Y, Xiu Y, Li B, et al (2011) Differential diagnostic value of single-photon emission computed tomography/spiral computed tomography with Tc-99m-methylene diphosphonate in patients with spinal lesions. Nucl Med Commun 32: 1194–1200. 10.1097/MNM.0b013e32834bd82e 21934544

[8] Pagenstert GI, Barg A, Leumann AG, Rasch H, Muller-Brand J, et al (2009) SPECT-CT imaging in degenerative joint disease of the foot and ankle. J Bone Joint Surg Br 91: 1191–1196. 10.1302/0301-620X.91B9.22570 19721045

[9] Schleich FS, Schurch M, Huellner MW, Hug U, von Wartburg U, et al (2012) Diagnostic and therapeutic impact of SPECT/CT in patients with unspecific pain of the hand and wrist. EJNMMI Res 2: 53 10.1186/2191-219X-2-53 23021154 PMC3506557

[10] Buchbender C, Sewerin P, Mattes-György K, Miese F, Wittsack H-J, et al (2013) Utility of combined high-resolution bone SPECT and MRI for the identification of rheumatoid arthritis patients with high-risk for erosive progression. Eur J Radiol 82: 374–379. 10.1016/j.ejrad.2012.10.011 23181974

[11] Khan KM, Tress BW, Hare WS, Wark JD (1998) Treat the patient, not the X-ray: advances in diagnostic imaging do not replace the need for clinical interpretation. Clin J Sport Med 8: 1–4. 9448948

[12] Huellner MW, Bürkert A, Strobel K, Lago MdSP, Werner L, et al (2013) Imaging non-specific wrist pain: interobserver agreement and diagnostic accuracy of SPECT/CT, MRI, CT, bone scan and plain radiographs. PLoS One 8: e85359 10.1371/journal.pone.0085359 24386468 PMC3875572

[13] Nathan M, Mohan H, Vijayanathan S, Fogelman I, Gnanasegaran G (2012) The role of ^99m^Tc-diphosphonate bone SPECT/CT in the ankle and foot. Nucl Med Commun 33: 799–807. 10.1097/MNM.0b013e328355880b 22692578

[14] Mohan HK, Gnanasegaran G, Vijayanathan S, Fogelman I (2010) SPECT/CT in imaging foot and ankle pathology-the demise of other coregistration techniques. Semin Nucl Med 40: 41–51. 10.1053/j.semnuclmed.2009.08.004 19958849

[15] Hirschmann MT, Davda K, Rasch H, Arnold MP, Friederich NF (2011) Clinical value of combined single photon emission computerized tomography and conventional computer tomography (SPECT/CT) in sports medicine. Sports Med Arthrosc 19: 174–181. 10.1097/JSA.0b013e3181ec8707 21540716

[16] Schwartz Z, Shani J, Soskolne WA, Touma H, Amir D, et al (1993) Uptake and biodistribution of technetium-99m-MDP during rat tibial bone repair. J Nucl Med 34: 104–108. 8418249

[17] Yildirim M, Gursoy R, Varoglu E, Oztasyonar Y, Cogalgil S (2004) ^99m^Tc-MDP bone SPECT in evaluation of the knee in asymptomatic soccer players. Br J Sports Med 38: 15–18. 14751939 10.1136/bjsm.2002.000695 PMC1724727

[18] Chung H, Kim Y, Hong S, Kim S, Chung J-K, et al (2000) Indirect signs of anterior cruciate ligament injury on SPET: comparison with MRI and arthroscopy. Nucl Med Commun 21: 651–658. 10994669 10.1097/00006231-200007000-00009

[19] Christensen SB (1983) Localization of bone-seeking agents in developing, experimentally induced osteoarthritis in the knee joint of the rabbit. Scand J Rheumatol 12: 343–349. 6658397 10.3109/03009748309099738

[20] Hart A, Buscombe J, Malone A, Dowd G (2005) Assessment of osteoarthritis after reconstruction of the anterior cruciate ligament: A study using single-photon emission computed tomography at ten years. J Bone Joint Surg Br 87: 1483–1487. 16260663 10.1302/0301-620X.87B11.16138

[21] Kim H, So Y, Moon S, Lee I, Lee S (2008) Clinical value of ^99m^Tc-methylene diphosphonate (MDP) bone single photon emission computed tomography (SPECT) in patients with knee osteoarthritis. Osteoarthritis Cartilage 16: 212–218. 17662626 10.1016/j.joca.2007.05.025

[22] Koike Y, Sano H, Kita A, Itoi E (2013) Symptomatic rotator cuff tears show higher radioisotope uptake on bone scintigraphy compared with asymptomatic tears. Am J Sports Med 41: 2028–2033. 10.1177/0363546513494741 23835267

[23] Wiewiorski M, Pagenstert G, Rasch H, Jacob AL, Valderrabano V (2011) Pain in osteochondral lesions. Foot Ankle Spec 4: 92–99. 10.1177/1938640010395749 21321364

[24] Kretzschmar M, Wiewiorski M, Rasch H, Jacob AL, Bilecen D, et al (2011) ^99m^Tc-DPD-SPECT/CT predicts the outcome of imaging-guided diagnostic anaesthetic injections: a prospective cohort study. Eur J Radiol 80: e410–415. 10.1016/j.ejrad.2010.09.013 20951520

[25] Pneumaticos SG, Chatziioannou SN, Hipp JA, Moore WH, Esses SI (2006) Low back pain: Prediction of short term outcome of facet joint injection with bone scintigraphy. Radiology 238: 693–698. 16436824 10.1148/radiol.2382041930

[26] Holder LE, Machin JL, Asdourian PL, Links JM, Sexton CC (1995) Planar and high-resolution SPECT bone imaging in the diagnosis of facet syndrome. J Nucl Med 36: 37–44. 7799079

[27] Dolan AL, Ryan PJ, Arden NK, Stratton R, Wedley JR, et al (1996) The value of SPECT scans in identifying back pain likely to benefit from facet joint injection. Br J Rheumatol 35: 1269–1273. 9010055 10.1093/rheumatology/35.12.1269

[28] Chicklore S, Gnanasegaran G, Vijayanathan S, Fogelman I (2013) Potential role of multislice SPECT/CT in impingement syndrome and soft-tissue pathology of the ankle and foot. Nucl Med Commun 34: 130–139. 10.1097/MNM.0b013e32835c0964 23211997

[29] Lee JW, Lee HY, Oh SW, Kim SK, Jeong KW, et al (2007) Evaluation of Usefulness of Radio-iodine SPECT/CT in Differentiated Thyroid Cancer. Nucl Med Mol Imaging 41: 350–358.

[30] Knupp M, Pagenstert GI, Barg A, Bolliger L, Easley ME, et al (2009) SPECT-CT compared with conventional imaging modalities for the assessment of the varus and valgus malaligned hindfoot. J Orthop Res 27: 1461–1466. 10.1002/jor.20922 19472383

[31] Meftah M, Katchis SD, Scharf SC, Mintz DN, Klein DA, et al (2011) SPECT/CT in the management of osteochondral lesions of the talus. Foot Ankle Int 32: 233–238. 10.3113/FAI.2011.0233 21477540

[32] Biswas D, Bible JE, Bohan M, Simpson AK, Whang PG, et al (2009) Radiation exposure from musculoskeletal computerized tomographic scans. J Bone Joint Surg Am 91: 1882–1889. 10.2106/JBJS.H.01199 19651945

[33] Sormaala MJ, Ruohola J-P, Mattila VM, Koskinen SK, Pihlajamäki HK (2011) Comparison of 1.5 T and 3T MRI scanners in evaluation of acute bone stress in the foot. BMC Musculoskelet Disord 12: 128 10.1186/1471-2474-12-128 21645348 PMC3121660

