# Supplementary figures and images for: Comparison of SPECT/CT and MRI in Diagnosing Symptomatic Lesions in Ankle and Foot Pain Patients: Diagnostic Performance and Relation to Lesion Type
Source: PLoS One. 2015 Feb 10;10(2):e0117583. doi: 10.1371/journal.pone.0117583 (PMC4323343; doi:10.1371/journal.pone.0117583)

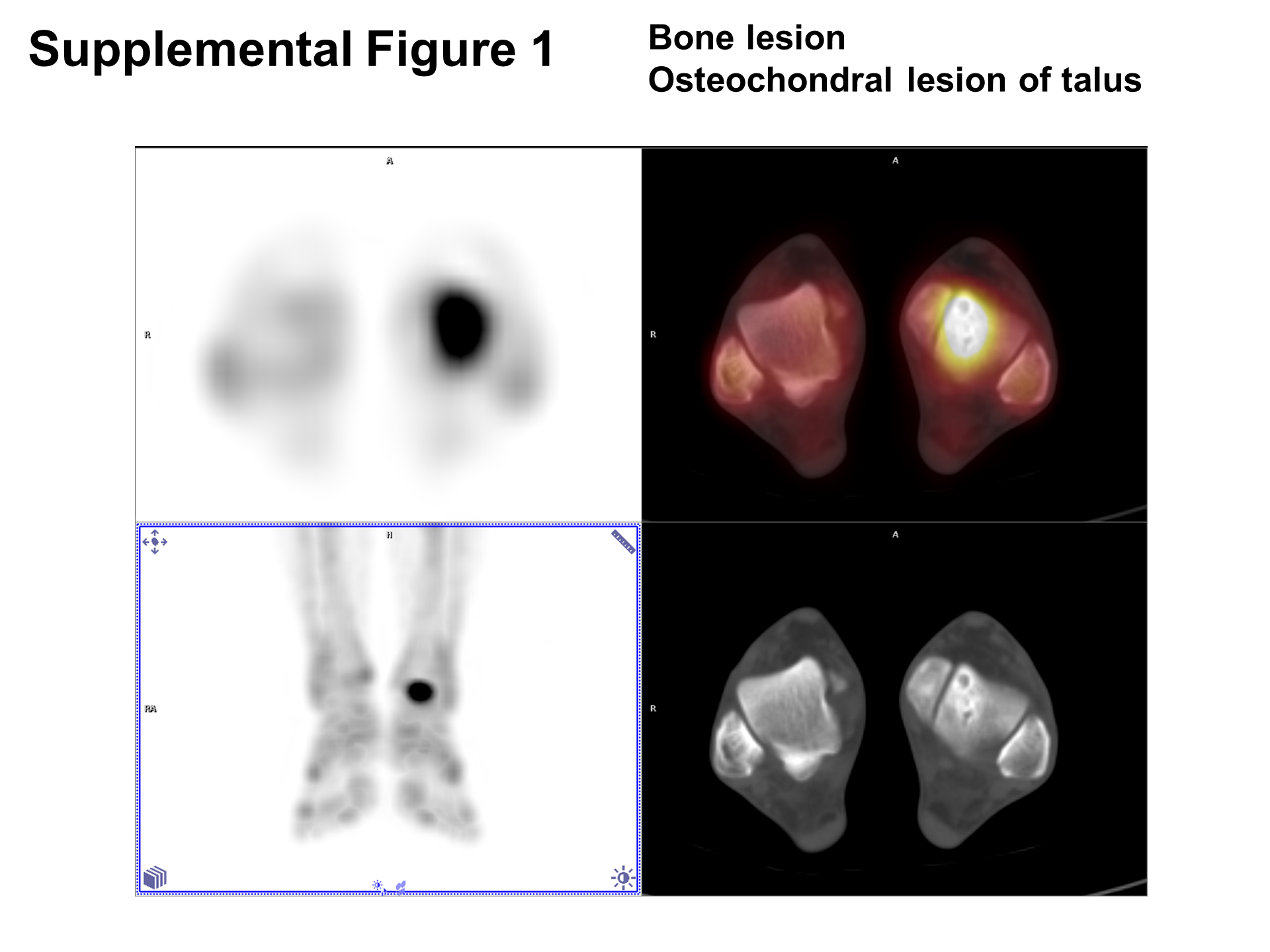

Supplement: S1 Fig — (TIF) [file pone.0117583.s001.TIF]

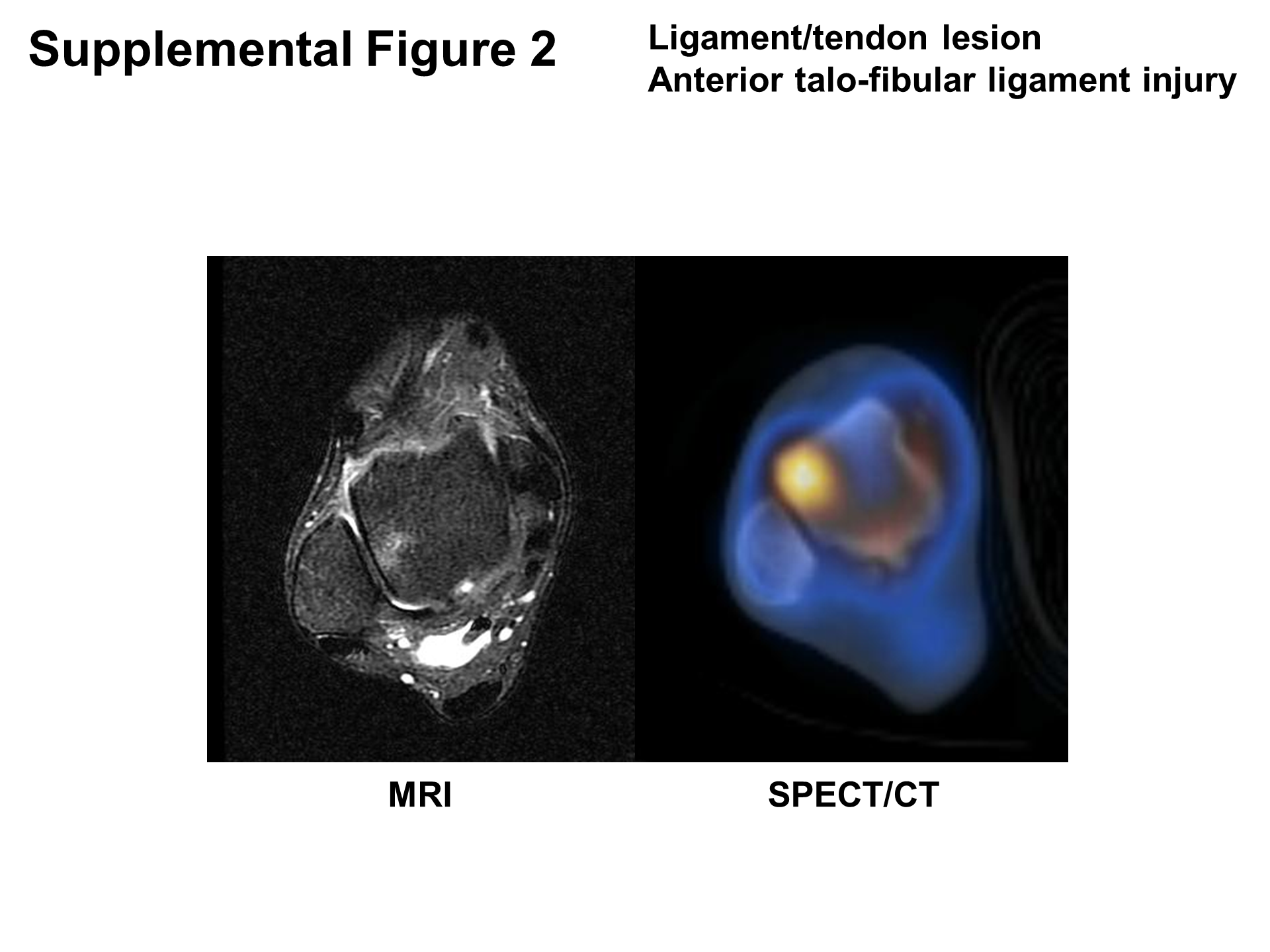

Supplement: S2 Fig — (TIF) [file pone.0117583.s002.TIF]

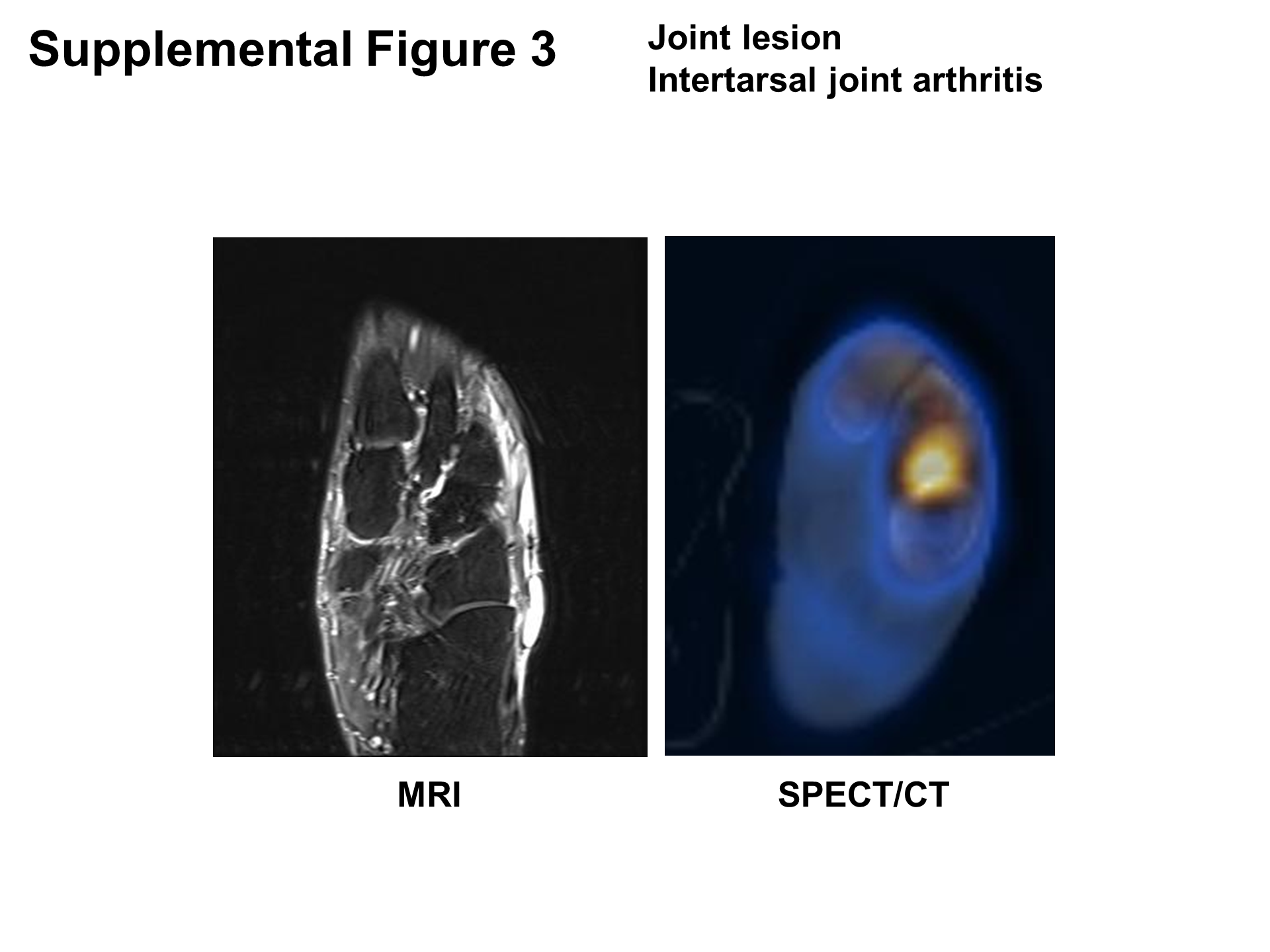

Supplement: S3 Fig — (TIF) [file pone.0117583.s003.TIF]
